# Hypoglycaemia in type 1 diabetes: technological treatments, their limitations and the place of psychology

**DOI:** 10.1007/s00125-018-4566-6

**Published:** 2018-02-08

**Authors:** Pratik Choudhary, Stephanie A. Amiel

**Affiliations:** 1 Diabetes Research Group, School of Life Course Sciences, Weston Education Centre, King’s College London, 10 Cutcombe Road, London, SE5 9RJ UK; 2 Department of Diabetes, King’s College Hospital Foundation Trust, London, UK

**Keywords:** Continuous glucose monitoring, Continuous subcutaneous insulin infusion, Diabetes technologies, Hypoglycaemia, Insulin analogues, Insulin pumps, Psychology, Review

## Abstract

Advances in technology allowing improved insulin delivery and glucose monitoring can significantly reduce the burden of hypoglycaemia when used appropriately. However, limitations of the current technology, and the skills, commitment and motivation required to use them, mean that it does not work for all people. Education and informed professional support are key to success. In the context of problematic hypoglycaemia, data suggest that newer technology has lower efficacy and uptake in those with most need. Identifying the causes of hypoglycaemia and understanding some of the underlying behavioural drivers may prove useful and psycho-educational strategies may be effective in selected individuals. Ultimately, as in many spheres of medicine, successful management of problematic hypoglycaemia depends upon matching the right treatment to the right individual.

**Electronic supplementary material:**

The online version of this article (10.1007/s00125-018-4566-6) contains a slide of the figure for download, which is available to authorised users.

## Introduction

Management of type 1 diabetes is complex, with patients juggling the competing risks of hyperglycaemia and hypoglycaemia. To achieve optimal glucose control, people with type 1 diabetes must adjust insulin dosing for predicted requirements, which vary according to food and alcohol intake, exercise, illness and other variables. The slow onset and long duration of action of currently available subcutaneous injections of insulin add to this challenge. Achieving ‘optimal’ glucose control is an uphill task that most people living with type 1 diabetes struggle to achieve. Hypoglycaemia and the fear it causes make a significant contribution to the higher than desired glucose results seen in national audits and registries [1–3].

Although the Diabetes Control and Complications Trial (DCCT) reported a threefold increased risk of severe hypoglycaemia (episodes requiring third-party assistance) with intensive insulin therapy [4] and a curvilinear relationship between severe hypoglycaemia and HbA_1c_, more recent observational data fail to confirm these findings [5]. Raised HbA_1c_ does not protect against hypoglycaemia, neither does lower HbA_1c_ necessarily increase its incidence. Age, duration of diabetes, previous occurrence of severe hypoglycaemia, impaired awareness of hypoglycaemia (IAH), C-peptide deficiency and lower socioeconomic status are key risk factors for severe hypoglycaemia [5]. Rates of severe hypoglycaemia in adults with type 1 diabetes are quoted at around 1.3 episodes per person per year and an estimated 18–36% of people with type 1 diabetes experience an episode in any one year; however, a small proportion of individuals experience a very high frequency of severe hypoglycaemia events [6, 7]. Recurrent exposure to mild biochemical hypoglycaemia reduces symptom awareness and counter-regulatory hormonal protection against hypoglycaemia [8] and can lead to IAH, with a three- to sixfold increased risk of severe hypoglycaemia [9, 10] and an impaired quality of life [11]. Although meticulous avoidance of hypoglycaemia has resulted in restored awareness in some studies [9], achieving this in routine clinical practice can be challenging.

Fear is an important motivator in diabetes self-management. Previous experience of hypoglycaemia and fear of hypoglycaemia remain limiting factors for many in achieving optimal glucose control [11, 12] and can lead to behaviours such as keeping glucose levels high or snacking to avoid hypoglycaemia [12]. On the other hand, fear of complications can act as a driver for people with type 1 diabetes to strive for very tight glucose control, which may be at the expense of frequent and significant hypoglycaemia [13].

## Impact of newer technologies

### Newer insulins

Use of analogue insulins (reviewed in [14]) has been associated with reduced risk of hypoglycaemia. Rapid-acting analogues, with faster onset and shortened duration of action, demonstrated a 20% reduction in the risk of severe hypoglycaemia [15]. Recently introduced ultra-fast-acting analogues may reduce this risk further [16]. Longer-acting basal insulins with less day-to-day variability and reduced nocturnal peak action have been associated with a reduced occurrence of nocturnal hypoglycaemia [17–19]. An impact on severe hypoglycaemia has not been consistently shown but many studies have excluded individuals with problematic hypoglycaemia. These factors may explain why rates of severe hypoglycaemia in observational ‘real-world’ studies are higher than those seen in randomised trials [20]. One exception was a randomised trial in individuals with IAH wherein an insulin analogue-based regimen produced a 29% reduction in the number of episodes of severe hypoglycaemia per person-year when compared with a regimen of soluble (regular) and NPH (intermediate-acting) insulin [21].

### Insulin pump therapy

Continuous subcutaneous insulin infusion (CSII) [22, 23] via an insulin pump allows insulin delivery to be varied according to the time of day. There have been many observational studies of CSII in individuals with suboptimal glucose control or who experience problematic hypoglycaemia despite multiple daily injections (MDI) of insulin. A meta-analysis of 19 studies comparing MDI with CSII found similar rates of severe hypoglycaemia between the two groups [24]. Slightly greater improvements in HbA_1c_ were seen with CSII—the greater the baseline value, the greater the benefit. Another meta-analysis examined studies in which participants receiving insulin by MDI had higher baseline rates of severe hypoglycaemia (>0.1 episodes per person-year, still less than the reported mean rate of severe hypoglycaemia): a 4.2-fold reduction in severe hypoglycaemia incidence was seen for CSII vs MDI [25]. Many of the studies included in these meta-analyses pre-dated modern ‘peakless’ basal insulins and most had no control arm and did not account for the educational input around starting pump therapy. However, observational data continue to demonstrate sustained reductions in the rate of severe hypoglycaemia in individuals starting CSII because of hypoglycaemia [26, 27].

The recently reported Relative Effectiveness of Pumps Over MDI and Structured Education (REPOSE) trial is the largest and longest RCT of CSII in type 1 diabetes [28]. Critically, this trial involved individuals who did not express a strong desire to use CSII and who did not have a specific indication or a need for a pump in the opinion of the investigators. Participants received structured education and were randomised to receive insulin by either CSII or MDI. Both groups showed equivalent improvements in HbA_1c_ and severe hypoglycaemia, the incidence of the latter being reduced by about half. These data support current guidelines advocating structured education in flexible insulin self-management before progression to CSII in the treatment pathway for problematic hypoglycaemia [29]. Where resources are limited, this sequence should ensure that available technology is focused initially on those with greatest need.

### Continuous glucose monitoring

Continuous glucose monitoring (CGM) provides information about the direction and rate of change of glucose and provides alarms to warn of impending hypoglycaemia. It is surprising that early studies of CGM failed to demonstrate a reduction in the incidence of hypoglycaemia [30–32]. This may have been related to very low baseline rates of hypoglycaemia, perhaps again driven by safety concerns in regulatory studies. A patient-level data meta-analysis was more encouraging [33] and later studies reviewed below have been more positive.

We refer here to open or real-time CGM, where the data are visible to the user and available for immediate response. Anecdotally, blinded CGM has been used to look at overnight glucose control and for educational purposes. One study failed to find any difference in hypoglycaemia detected by blinded CGM between people with and without intact awareness of hypoglycaemia [10]; this unexpected finding may relate to the short period of data collection.

As with CSII, the importance of education cannot be underestimated. The Multicenter 2 × 2 Factorial Randomized Controlled Trial Comparing Insulin Pump with Multiple Daily Injections and Continuous with Conventional Glucose Self-monitoring (HypoCOMPaSS) was one of the largest RCTs of diabetes technologies in those with IAH. It randomised 96 people with type 1 diabetes and with IAH in a 2 × 2 fashion to receive CGM or not and CSII or not [34]. Although the number of episodes of severe hypoglycaemia per person-year was very high at baseline and fell dramatically throughout the study, it did not differ between the four randomised groups. All participants received a short group education package and weekly support from researchers during the 24 weeks of the main study and the global benefit has been attributed to this. On the other hand, the IN CONTROL study [35], which also recruited people with IAH, demonstrated significant reductions in the rates of severe and biochemical hypoglycaemia in participants who used CGM but who had received no intensive education. Only 35% of participants used carbohydrate counting, a marker for appropriate current diabetes education, and under half of the participants used CSII. Notably, the improvement was lost after participants crossed over to their previous therapy, thus indicating no evidence of any learning effect. Two recent studies of CGM in individuals using MDI demonstrated significant reductions in the duration of hypoglycaemia (by almost half) but rates of severe hypoglycaemia were low at baseline [36, 37] .

Sensor-augmented pumps (SAPs) can suspend insulin delivery for actual or predicted hypoglycaemia [38, 39]. One RCT, which compared a device providing automated suspension of insulin delivery in response to sensor hypoglycaemia vs SAP without this feature, demonstrated a 38% reduction in the number of nocturnal hypoglycaemic events [40]. There was no episode of severe hypoglycaemia in this trial. In the same year, Ly et al [41] reported a significant reduction in the incidence of moderate and severe hypoglycaemia using the automated threshold suspend system against CSII in 95 children and young adults.

Intermittently monitored, retrospective CGM, or ‘flash’ monitoring, provides easily accessible glucose data, including directional trends but no alarms or alerts for hypoglycaemia. Obviating the need for finger prick should improve rates of monitoring and control but this remains to be proven. An RCT in individuals with tight glucose control reported a 38% reduction in sensor-detected glucose values under 3.9 mmol/l, with no deterioration in overall glucose control, but this study excluded those with IAH or previous severe hypoglycaemia [42]. A recent study in people with IAH found no impact on hypoglycaemia, compared with real-time CGM [43], and further studies are needed.

Hypoglycaemia is also an issue in insulin-treated type 2 diabetes, but the use of technology to prevent hypoglycaemia in this group has not been studied in detail. An RCT of CSII vs MDI in type 2 diabetes (OPT2MISE) did show CSII to be of benefit in those who were unable to improve control despite high doses of insulin, although the incidence of hypoglycaemia was low in both groups [44].

A common contributor to hypoglycaemia is over-correction of high glucose values, which result from repeated injections of rapid-acting insulin within a short period of time causing overlapping or ‘stacking’ of insulin doses. This is particularly important for individuals using CGM or ‘flash’ monitoring, whereby they see rapid fluctuations in glucose and/or receive alerts about glucose measurements that may be out of range. Software that can perform the calculation of insulin dose, while accounting for the ‘insulin action on board’, has been available in insulin pumps for a while. Greater use of the bolus advisor software on pumps has been associated with better control of postprandial glucose and a non-significant reduction in the frequency of postprandial hypoglycaemic events [45]. Conclusive data are not yet available, although a meta-analysis of six small studies of bolus advisors used with insulin pumps found a non-significant reduction in the number of hypoglycaemic episodes [46]. Incorporation of such technology into capillary plasma glucose meters and smartphone health applications has improved glucose control and reduced glucose variability, although no consistent impact on hypoglycaemia has been reported [47–50]. Some studies have demonstrated reduced fear of hypoglycaemia with the use of these devices [51] and many people with diabetes welcome their use. However, some individuals still express reservations and are reluctant to use automated advice (especially on basal insulin adjustment) without understanding how that advice was generated [52]. There are important caveats to the use of smartphone health applications: many have not been tested as rigorously as other medical technologies and may carry risk of inappropriate dose advice [53].

Despite these advances, we have not yet reached a Utopia wherein all have access to and are able to use the new technologies reliably to achieve optimum glucose control with minimal or no hypoglycaemia. Notably, while studies have demonstrated reduction in the number of episodes of severe hypoglycaemia with CGM, the benefit only persists while sensors are being used. None of the technologies have been shown to restore subjective awareness of hypoglycaemia or the impaired counter-regulatory responses of IAH needed for endogenous protection against severe hypoglycaemia. ‘Technological awareness’ only functions when the sensors are worn. The evidence to date thus supports a step-wise approach to the management of problematic hypoglycaemia, starting with validated structured education programmes transferring the skills of insulin dose adjustment to the users of the insulin, and progressing through use of technology, either pump or sensor, then SAP therapy with automated suspend features. If these conventional measures fail, where available, islet or pancreas transplantation may be an option [29]. Using this algorithm, by progressing through the steps every 3–6 months and being supported throughout by an experienced multi-disciplinary healthcare team, we have been able to alleviate problems with recurrent severe hypoglycaemia in most of the individuals affected [54]. It is unclear exactly where interventions such as flash glucose monitoring and closed-loop systems fit into this algorithm, as they have not been tested in this population; however, they are likely to provide a further level of protection. In future, a readily available fully closed-loop ‘artificial pancreas’ may change this algorithm if the device is able to maintain normoglycaemia without hypoglycaemia and proves acceptable to all users.

## Limitations of technology

### Healthcare professional factors

Appropriate selection of candidates for treatment with new technologies is key to success, as is familiarity of healthcare professionals with the therapies. Emerging data suggest that a positive attitude on the part of healthcare professionals, combined with structured education, is vital for patient uptake. Healthcare professionals’ attitude and knowledge are especially important for technologies such as CSII and CGM [55] that require a degree of technological competence and, through the additional opportunities and information they provide, can increase demands on patients when compared with MDI and intermittent capillary glucose monitoring. There is some evidence for a systematic bias in the way patients are selected for these therapies and some individuals who may stand to gain may be being denied the opportunity to use them [55]. Inexperience in providing support to users of newer technologies can be a barrier to successful implementation and a non-physiological insulin regimen may ensue, with its attendant risks. With CGM, recommendations on how to respond to direction trends [56] are not widely tested, standardised or followed. One published algorithm with some evidence of benefit is presented in the Diamond study [37]. Greater experience may underpin the successes reported by larger centres [57] and there is a need for evidence-based guidelines to inform the whole community.

### User factors

Any new piece of technology is only as good as the person using it. Patients need to use and interact appropriately with the equipment, incorporating tasks such as regularly changing sensors and infusion sites and calibrating sensors. Different CGM systems have different requirements but many depend on appropriate timing of calibrations, even as the frequency with which these must be done diminishes with increasing sophistication. An important aspect of this newer technology is the ability to use the data to obtain benefit—looking frequently at sensor glucose values and making appropriate decisions, based on immediate information and on trends in the data. This can require more thought and work and can sometimes be overwhelming.

### Education

We have described how structured education programmes that teach self-management of flexible insulin regimens are at least as good in their impact on severe hypoglycaemia as technological solutions (reviewed in this issue of Diabetologia), demonstrating up to 50% reduction in the rates of severe hypoglycaemia [58]. In addition, some of these programmes have shown restoration of awareness of hypoglycaemia in up to 40% of individuals who reported IAH at baseline [59, 60]. Nevertheless, audit data still show that many graduates of these programmes do not achieve optimal glucose control [2, 59–62]. For these people the newer technologies should be considered sooner rather than later.

### Treatment adherence

Not all eligible individuals will take to new technology. While most diabetic individuals report improved quality of life with CSII [63], and discontinuation rates for CSII are low, discontinuation does occur. Not enough has been done to understand the reasons behind discontinuation of CSII. In one study of young people who stopped pump therapy after some years, reasons included a greater sense of disease (93%), difficulties during sports (70%), a feeling of worsened wellbeing (63%), having to attach the pump to the body (60%) and embarrassment (56%) and pain (50%) during needle insertion [64].

As with any therapy, not everyone gains full benefit. In a study of 463 children and adolescents starting CSII, while the overall frequency of severe hypoglycaemia reduced from 14.3 to 3.3 episodes per 100 person-years (*p* < 0.001), 20% did not respond despite good adherence [65]. It is also important to recognise that technology is not universally available. Remaining with the paediatric literature, we see that CSII use is more common in those from higher parental education groups and higher annual household income [66].

For some people, defective cutaneous absorption of insulin is a hypothesised barrier to stable glucose control. Intraportal insulin delivery via continuous intraperitoneal insulin infusion (CIPII) has demonstrated significant reductions in rates of severe hypoglycaemia when compared with CSII [67]. This administration system is not widely available and complications have included port infections (0.5 events per person-year) and abdominal pain (0.2 events per person-year) [67].

Sensor use remains problematic for reasons that are not fully understood. Occasionally, individuals may use the added information to drive glucose levels lower and real or perceived inaccuracies undermine trust. The INTERPRET study [68], published in 2013 and using the technology available at the time, followed 263 individuals in 15 countries who started SAP (8% due to problematic hypoglycaemia). Only four study participants stopped using the CSII, but average sensor use was 30% and decreased with time. During this study, nine patients reported 13 episodes of severe hypoglycaemia, with four episodes requiring hospitalisation; there were no reported events in the year prior to the study. Despite this, the perceived frequency of hypoglycaemia was reduced, as were behaviours and worries about hypoglycaemia, showing the importance of measuring all outcomes.

There is a well-recognised relationship between frequency of conventional blood glucose measurements and glucose control [69, 70]. Similarly, For CGM to be associated with HbA_1c_ benefit, sensors must be worn for at least 70% of the time [33] and, anecdotally, severe hypoglycaemia occurs in sensor users when the sensors are not worn. Similar relationships are seen with frequency of bolusing and cannula changes in pump users [65, 69, 70]. Age may be a factor in usage. The JDRF–CGM study found a much lower adherence in young adults [31], although this was not noted in later studies [40, 71]. As the technologies develop, and experience with them grows, treatment adherence has risen from around 60% [30, 31, 72] to above 90% [32, 36, 37, 71], accompanied by reductions in mild biochemical hypoglycaemia [36, 37, 71]. However, avoidance of biochemical hypoglycaemia is not necessarily associated with improvement in IAH [34].

Outside research studies, the type 1 diabetes exchange registry data show that only 9% of patients were using CGM and 41% of users had stopped using CGM over the last year [73]. In a representative paediatric cohort, various reasons for discontinuing CGM were given: discomfort while wearing the sensor (42%), problems with inserting the sensor (33%), problems with adhesives (30%), too many alarms (27%) and concerns about accuracy (25%) [69]. In the SENLOCOR study, an observational study of individuals using CGM, although there was a fall in HbA_1c_ levels from 8.2% to 7.7% (66 to 61 mmol/mol) and a reduction in the proportion of patients experiencing severe hypoglycaemia from 20% to 13.6%, the time spent using CGM reduced from 86% at 3 months to 68.9% at 6 months [74].

### Accuracy

Managing expectations of the performance of technology is key. In silico modelling suggests that there is increased occurrence of hypoglycaemia with increased glucose sensor error; an abrupt slope change at a mean absolute relative difference (MARD) between sensor and blood glucose of 10% [75]. Some of the concerns around accuracy may arise from the physiological lag between blood and interstitial glucose. This lag is between 3 and 10 min, and can be greater when glucose concentrations are changing rapidly [76, 77]. During a rapid fall in blood glucose levels prior to hypoglycaemia, sensor glucose readings may be 1–1.5 mmol/l higher than actual blood glucose, meaning that the user may be reassured by a sensor glucose reading of 4 or 4.5 mmol/l while blood glucose could be 3.0 mmol/l or lower. Such experiences may militate against sensor use. The MARD between sensor and blood glucose has fallen from around 20% in early CGM systems to just over 10% for most currently available systems [78]. Studies with the newer, more accurate devices, have shown higher levels of adherence to use [37, 71].

### Alarm fatigue

**Fig. 1:**
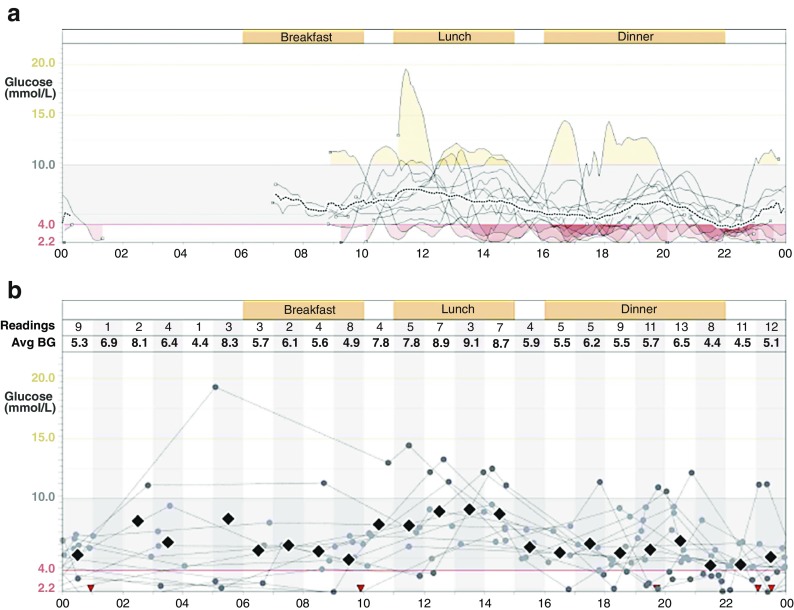
(**a**) Screenshot from a download of data from a continuous glucose sensor belonging to a woman with long-duration type 1 diabetes who had IAH and recurrent severe hypoglycaemia (one of our patients at King’s College Hospital), using Medtronic Carelink software (www.medtronicdiabetes.com/products/carelink-personal-diabetes-software). The SAP featured automated threshold suspend but the download shows that the sensor was not being used during the night (absence of sensor tracing overnight) because of the user’s frustrations with alarms overnight. (**b**) Data from capillary glucose monitoring (the circles seen through the night) in the same individual over the same time confirm that the patient was indeed continuing to experience recurrent biochemical hypoglycaemia (below 4 mmol/l) during the night. This patient had declined advice on multiple occasions to reduce overnight basal insulin. Avg BG, average blood glucose. This figure is available as a downloadable slide

CGM requires the user to respond to alerts and alarms. At night, alarms may disturb partners more than the person wearing the device, with the latter often staying asleep and failing to respond appropriately to the alarm [79]. The alarm is a constant reminder that glucose levels are not in range and many individuals give ‘alarm fatigue’ as a reason for discontinuing sensor use [69, 70, 73]. Anecdotally, CGM users complain about the frequency of alarms even while recognising that those alarms are appropriate and that they require a response (Fig. 1). In contrast, responding to a predictive alarm can result in eating carbohydrates even though the insulin pump is suspended, with resultant hyperglycaemia. Home users of early closed-loop CGM systems reported many positive features, including greater reassurance around overnight glucose control, reduced worry and better functioning during the day after improved glucose control and sleep; they also reported frustrations with alarms, technical difficulties and the size of the equipment [80]. Some of these concerns may recede as the technology improves but we should recognise that early adopters and people volunteering for research with new devices are likely to be more motivated than other potential users. Provision of training and support in the use of CGM is key to reducing anxiety and encouraging effective use.

## The psychological associations of IAH

Inevitably there are some diabetic individuals who seem unable to gain benefit from technology. In the context of this review, we focus on those with problematic hypoglycaemia, in whom the lack of benefit may be partly explained by the central mechanisms responsible for IAH. Neuroimaging data have led to suggestions that those with IAH may not perceive hypoglycaemia to be stressful and unpleasant. This may lead to reduced internal motivation to avoid hypoglycaemia. In a study of adults with type 1 diabetes in a specialist clinic in Sweden, Anderbro and colleagues found 8% of participants to be at high risk of severe hypoglycaemia but who were relatively unconcerned about this [81]. In another report, when compared with individuals with normal awareness of hypoglycaemia in a UK clinic, those individuals with IAH were significantly less likely to follow advice on insulin regimen adjustment, presumably often focusing on hypoglycaemia avoidance [82]. Detailed interviews with individuals with IAH reveal that many of them do not see severe hypoglycaemia as a major problem, or at least see even mildly raised glucose levels as far more serious [83, 84]. Fear of hyperglycaemia may underlie failure of attempts to avoid hypoglycaemia. It is often family members who are far more distressed about a person’s hypoglycaemia [84].

## Future directions

Some of the barriers to adoption of new technology may resolve as the apparatus improves. Perfection of closed-loop insulin delivery should reduce the need for alarms and remove frustrations related to equipment failure or intrusiveness. Anxieties about computer-generated advice on insulin dosing may be allayed by greater understanding of how the algorithms work. Psychological support may be required to quell fears of becoming dependent on external equipment and dislike of being attached to machinery that provides a constant reminder of having type 1 diabetes and the attendant personal vulnerability. There is early evidence that addressing health beliefs around hypoglycaemia that have created barriers to hypoglycaemia avoidance may help those most prone to problematic hypoglycaemia to gain benefit from existing interventions and technologies, thus reducing their hypoglycaemia experience and restoring their (and their family’s) quality of life [85].

Ensuring adequate provision of evidence-based and informed education and, where indicated, psychological support, around use of both conventional and new technologies will help achieve best and most cost-effective outcomes for more people living with the demands of managing type 1 diabetes and ensure that the expected benefits of the new technologies can be realised.

## Electronic supplementary material

ESM Downloadable slide (PPTX 147 kb)

## Abbreviations

CGM: Continuous glucose monitoring
CSII: Continuous subcutaneous insulin infusion
HCP: Healthcare professional
IAH: Impaired awareness of hypoglycaemia
MARD: Mean absolute relative difference
MDI: Multiple daily injection
SAP: Sensor-augmented pump
