# Supplementary material for: Hypoglycaemia in type 1 diabetes: technological treatments, their limitations and the place of psychology
Source: Diabetologia. 2018 Feb 8;61(4):761–9. doi: 10.1007/s00125-018-4566-6 (PMC6448988; doi:10.1007/s00125-018-4566-6)
Supplement: Supplementary file 1 — (PPTX 147 kb) [file 125_2018_4566_MOESM1_ESM.pptx]

## Slide 1
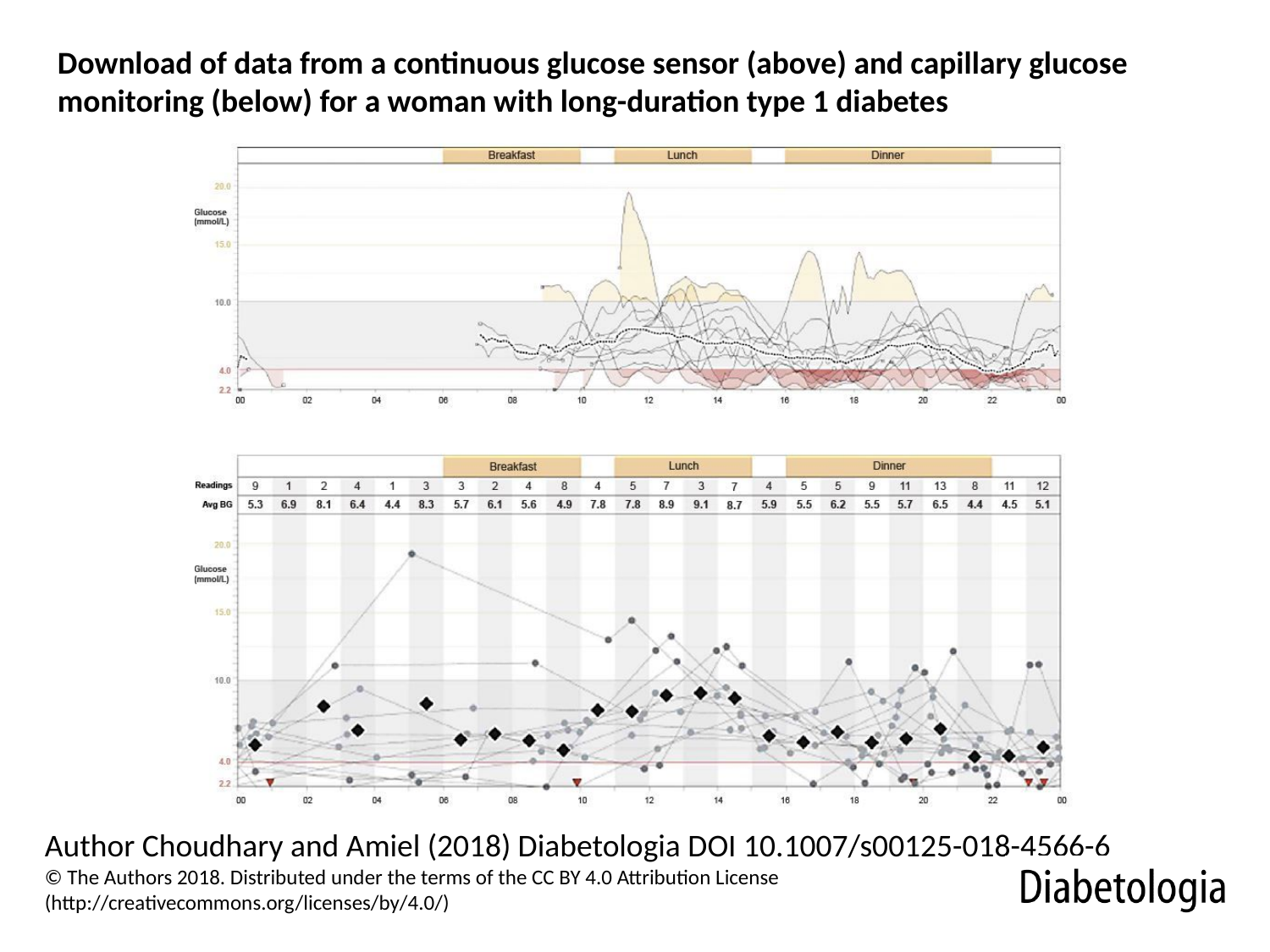

Download of data from a continuous glucose sensor (above) and capillary glucose monitoring (below) for a woman with long-duration type 1 diabetes
FIGURE HERE
Author Choudhary and Amiel (2018) Diabetologia DOI 10.1007/s00125-018-4566-6
© The Authors 2018. Distributed under the terms of the CC BY 4.0 Attribution License (http://creativecommons.org/licenses/by/4.0/)
